# A comparison of the upper lip bite test with hyomental/thyrosternal distances and mandible length in predicting difficulty in intubation: A prospective study

**DOI:** 10.4103/0019-5049.76603

**Published:** 2011

**Authors:** Zahid Hussain Khan, Anahid Maleki, Jalil Makarem, Mostafa Mohammadi, Ramooz Hussain Khan, Ali Zandieh

**Affiliations:** Department of Anesthesiology and Intensive Care, Imam Khomeini Medical Centre, Tehran University of Medical Sciences, Tehran, Iran; 1 Department of Dentistry, Debrecen Medical University, Hungary

**Keywords:** Difficult intubation, difficult laryngoscopy, endotracheal intubation, predictive airway tests

## Abstract

The incidence of difficulty in tracheal intubation has been reported to range from 0.5 to 18% in patients undergoing surgery. We aimed to elucidate the role of upper lip bite test (ULBT) with other prevailing tests, hyomental/thyrosternal distances (HMD/TSD), and the mandible length (ML) and their possible correlation in predicting difficulty in intubation. After institutional approval and informed consent were obtained, 300 consecutive patients aged 20–60 years of ASA physical status I and II, scheduled for elective surgical procedures requiring tracheal intubation and meeting the inclusion criteria, were enrolled in this study. Each patient was evaluated regarding ULBT, HMD, TSD and ML. Laryngoscopy was assessed by an attending anaesthesiologist blinded to the measurements. The laryngoscopic result was graded according to Cormack and Lehane’s Grading system. The negative predictive value (NPV) and positive predictive value (PPV) of ULBT were found to be 94 and 100%, respectively. These corresponding figures for TSD were 88.5 and 0%, respectively. Specificities for ULBT, HMD, ML and TSD were 100, 98.9, 98.9 and 98.1%, respectively. ULBT class and laryngoscopic grading showed the greatest agreement (kappa = 0.61, *P* < 0.001). An agreement between laryngoscopic grading and HMD and ML also existed (0.003 and <0.001, respectively), but was comparatively weaker. The high specificity, NPV, PPV and accuracy of ULBT as revealed in this study could be a good rationale for its application in the prediction of difficulty or easiness in intubation. ML > 9 cm and HMD > 3.5 cm were good predictors of negative difficult intubation.

## INTRODUCTION

There is an impelling need for accurate tests to predict difficult intubation, as failure to achieve endotracheal intubation causes morbidity and mortality in anaesthetised patients.[1] The paucity of fool proof tests in predicting difficult intubation commonly results in unanticipated difficult scenarios and their attendant repercussions. The reported incidence of difficult intubation ranges from 0.5 to 18%.[2–6] It is obvious and abundantly clear that preoperative identification of patients whose trachea will be difficult to intubate would decrease the rate of anaesthesia related adverse respiratory events. The upper lip bite test (ULBT) described by Khan *et al*.[7] has come under scrutiny these years. We have tried to elucidate the role of ULBT as a simple bedside airway predictive test with the other prevailing tests, the hyomental/thyrosternal distances (HMD, TSD), and the mandible length (ML) in predicting difficulty in endotracheal intubation. The hypothesis underlying this study was to compare the labiomandibular morphometry with cervicomandibular morphometry, and to test whether ULBT had a positive correlation with HMD, TSD, ML, and whether each of these parameters in turn had a direct correlation with difficult laryngoscopic view and difficult intubation.

## METHODS

After having obtained approval from our institution’s ethical committee and informed consent from the patients, 300 consecutive male and female patients of ASA physical status I or II, aged 20–60 years, scheduled to undergo elective surgery under general anaesthesia between July 2008 and June 2009 were considered for enrollment. All the procedures of the current study were conducted in accordance with Helsinki declaration. Edentulous patients, those unable to open the mouth, patients with pharyngolaryngeal pathology, with a history of thyroid or neck surgery, pregnancy, or with limitation of temporo-mandibular and atlanto-axial joints were excluded from the study.

Preoperatively, two senior residents not involved in intubating the airways they evaluated, assessed the ULBT class and obtained measurements of HMD, TSD, and ML. The ULBT class was determined according to the following criteria: class I, lower incisures can bite the upper lip above the vermilion line; class II, lower incisors can bite the upper lip below the vermilion line; and class III, lower incisors cannot bite the upper lip.[7] The HMD was measured in supine position with the head fully extended and with the mouth closed as the straight distance from the lower border of the mandibular mentum to the superior border of the hyoid bone in centimetres. The TSD was also measured in the supine position with the head fully extended and the mouth closed as the distance between prominentia laryngea of the thyroid cartilage and incisura jugularis of the sternal bone. ML was measured from the angle of mandible to the tip of the chin.[4] ULBT of class III, HMD < 3.5 cm, TSD < 6.5 cm, and ML< 9 cm were considered as markers of a potentially difficult intubation based on receiver operating characteristic (ROC) analysis.

After induction of anaesthesia, anaesthesiologists blinded to the preoperative measurements attempted laryngoscopy with a Macintosh No. 3 blade (Welch Allyn Inc., Seaneatills Falls, NY, USA) and determined the laryngoscopic view using the Cormack-Lehane (C–L) grading system,[8] as follows: grade I, full view of the glottis; grade II, glottis partly exposed, anterior commissure not seen; grade III, only epiglottis seen; grade IV, epiglottis not seen. No external laryngeal pressure was applied while reporting the laryngeal view. C–L grades I and II were considered as “easy intubations”, and grades III and IV as “difficult intubations”. Intubations were declared as difficult if a second anaesthesiologist failed to visualise the larynx using conventional measures of changing the blade, head position, and application of external laryngeal pressure. In such circumstances, a gum-elastic bougie, video laryngoscope, or a fibreoptic was considered.

Statistical analysis was performed using SPSS software version 16 (SPSS, Chicago, IL, USA). Data were analysed using kappa agreement and calculation of sensitivity, specificity, positive predictive value (PPV), negative predictive value (NPV), and accuracy with their 95% confidence interval (95% CI). Kappa values measuring agreement between tests were calculated. A P value less than 0.05 was considered significant. ROC was applied for determination of cut points.

## RESULTS

**Figure 1:**
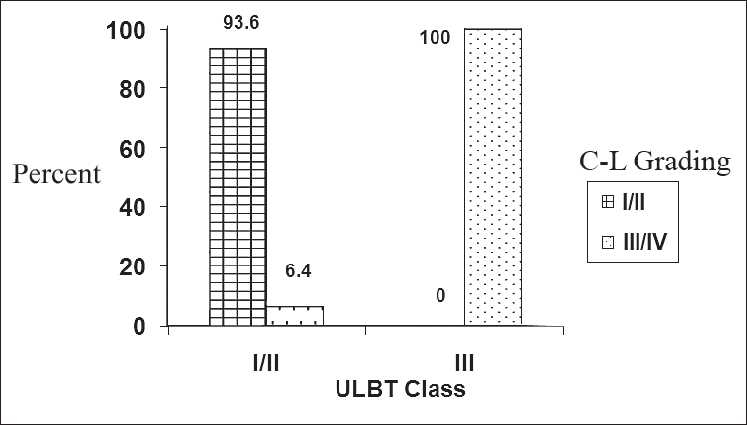
Frequency of laryngoscopic view (C– L grading) according to the ULBT class

Out of 300 patients recruited in this study, 125 (41.7%) were males. The mean age was 38.4 ± 9.2 years (mean ± SD). Difficult laryngoscopy (C–L grades III and IV) was seen in 38 (11.3%) patients, but a difficult intubation was encountered in none of the patients. There was no significant difference regarding difficult laryngoscopic view based on gender (*P* > 0.05). ULBT of class III was seen in 16 (5.3%), HMD < 3.5 cm in 6 (2%), TSD < 6.5 cm in 5 (1.7%) and ML < 9 cm was seen in 9 (3%) patients. Different classes of ULBT and measurements of other predictive tests versus C–L grades are depicted in Table 1. A significant agreement was found between ULBT, ML, and HMD and the laryngoscopic view (*P* < 0.001, *P* < 0.001 and *P* = 0.03, respectively), but such an agreement was absent between TSD and the laryngoscopic view [Table 1, Figure 1]. The greatest agreement seen was between ULBT and laryngoscopic view (kappa coefficient = 0.61). The specificity and NPV of all the predictive tests were high and ranged between 98.1 and 100% and 88.5 and 93.7%, respectively. Both specificity and NPV were the highest in ULBT in comparison with other predictive tests. ULBT had the highest sensitivity of 47.1% and it also had the highest PPV of 100%, reflecting that a higher class of ULBT could correctly predict a difficult intubation. TSD had the lowest parameters in comparison with the other tests [Table 2].

**Table 1:** Agreement of ULBT, HMD, TSD and ML with laryngoscopic view

		Laryngoscopic view		Kappa coefficient	*P* value
		I/II	III/IV		
ULBT	I/II	266 (88.7)	18 (6)	0.61	<0.001
	III	0	16 (5.3)		
HMD	≥3.5 cm	263 (87.7)	31 (10.3)	0.12	0.003
	<3.5 cm	3 (1)	3 (1)		
TSD	≥6.5 cm	261 (87)	34 (11.3)	0.03	0.42
	<6.5 cm	5 (1.7)	0		
ML	≥9 cm	263 (87.7)	28 (9.3)	0.24	<0.001
	<9 cm	3 (1)	6 (2)		

HMD/TSD, hyomental/thyrosternal distances; ML, mandible length; ULBT, upper lip bite test, Percent is calculated by dividing every cell number by 300, Figures in parentheses are in percentage

**Table 2:** Sensitivity, specificity, PPV, NPV and accuracy of ULBT, HMD, TSD and ML

	Sensitivity	Specificity	PPV	NPV	Accuracy
ULBT class III	47.1 (30.2–64.6)	100 (98.2–100)	100 (75.9–100)	93.7 (90–96.1)	94 (90.5–96.3)
HMD < 3.5 cm	8.8 (1.7–30.7)	98.9 (95.4–99.8)	50 (10–90)	89.5 (83.7–93.4)	88.7 (84.4–91.9)
TSD < 6.5 cm	0 (0–18.7)	98.1 (94.3–99.5)	0 (0–65)	88.5 (82.6–92.6)	87 (82.5–90.5)
ML < 9 cm	17.6 (5.8–40.8)	98.9 (95.4–99.8)	66.7 (24.3–93.5)	90.4 (84.8–94.1)	89.7 (85.5–92.8)

HMD/TSD, hyomental/thyrosternal distances; ML, mandible length; NPV, negative predictive value; PPV, positive predictive value; ULBT, upper lip bite test

## DISCUSSION

Difficult laryngoscopy and intubation ushers in irreparable damage to the patient if not handled quickly and diligently. Since no anatomical factor can correctly forecast a difficult intubation with 100% accuracy, we might expect predictive tests to be unreliable. Little work has been published regarding the use of HMD or TSD as screening tests to detect difficulty in intubation. This study was designed to evaluate the efficacy of ULBT, HMD, TSD, and ML in forecasting a difficult intubation, and to draw a possible correlation between the tests and C–L grades. Based on our maiden study[7] wherein the ULBT provided a high accuracy (Ac) and specificity, in this study, we compared ULBT with the other prevailing tests to assess the efficacy of labiomandibular morphometry and cervicomandibular morphometry in predicting difficulty in intubation. Although difficult laryngoscopic view was seen in 11.3% of the patients which corroborates with other reports,[2–6] we did not encounter any difficulty in intubation. As found in this study, 16 patients who had a ULBT of class III were all found to have a C–L grade of III or IV, reflecting a strongly positive correlation between a higher ULBT class and difficult laryngoscopic view. This point was further substantiated and statistically endorsed by a PPV of 100% which was found in this particular class of ULBT, i.e., class III. Again, the accuracy of ULBT was found to be higher than the other tests, which testifies that the ULBT carries lower false positive and negative values in predicting a difficult laryngoscopic view. In patients who had ULBT of class I or II, the probability of a difficult laryngoscopic view was exceedingly low which is in agreement with our earlier studies[7,9] reporting that ULBT class I and II could serve as valuable predictors of an easy laryngoscopic view. These findings are also supported by the highest NPV found for ULBT in our present study. All the other tests also had a high NPV, reflecting that they correlated well with the ease of laryngoscopy. Other tests also had comparatively high accuracy ranging from 87 to 94%. TSD failed to be of any help as a bedside test in predicting difficult intubation. Unlike the thyromental distance,[10–12] the TSD fails to take into account viewing the oropharynx and thus fails to provide any significant data regarding airway difficulty. As regards sensitivity and specificity, ULBT had the highest figures whereas TSD had the lowest figures. Compared to HMD and ML which had a significant agreement with laryngoscopic view, there was no agreement between TSD and laryngoscopic grading. In 87.7% of patients with HMD ≥ 3.5 cm, ULBT classes I and II were more frequent with concomitant C–L grades of I and II. On the contrary, HMD < 3.5 cm which was considered as a marker of difficult intubation moved in tandem with ULBT class III and C–L grade III/IV. Again, in 2% of patients with ML < 9 cm, ULBT class III and C–L grade III were common occurrences, meaning difficult intubation as hypothesised. These findings also suggest that both HMD and ML, if higher, serve as predictors of easy intubation as the tongue can be easily compressed into these spaces during laryngoscopy. Previously, it had been shown that HMD is a valuable predictor of difficult intubation.[13] Association of ML with difficult intubation was evaluated in a few studies, but no significant finding was reported.[14,15] TSD was associated with difficult intubation in monovariate analysis; however, it was not a significant predictor of difficult intubation in multivariate analysis.[16]

Owing to the latitude the ULBT provides in correctly assessing the labiomandibular morphometry coupled of course with temporo-mandibular joint excursion and taking into consideration buck teeth while the test is being conducted, ULBT proved effective as a simple, reliable predictive airway test in this study.

In conclusion, there is a stepwise increase in the incidence of C–L grade III and IV as the ULBT class shows a rise from I to II, and from II to III. A similar cascade of laryngoscopic view was noted as the ML and HMD decreased from their predetermined values of 3.5 and 9 cm, respectively. Compared to the other tests which are quantitative in nature, ULBT is an independent and qualitative parameter, thereby enhancing its potential value of being diagnostic in airway assessment.
